# Primary pyloroduodenal tuberculosis presenting as gastric outlet obstruction. A case report

**DOI:** 10.1016/j.ijscr.2024.109618

**Published:** 2024-04-06

**Authors:** Molla Asnake Kebede, Sisay Mengistu Mohammed, Dessalegn Woretaw Ayehu, Yilkal Teshome Numaro, Woineab Mengista Tadeg, Zerubabel Girma Tesso

**Affiliations:** a Department of Medicine, School of Medicine, College of Medicine and Health Sciences, Mizan Tepi University, Mizan-Teferi, Ethiopia; b Department of Surgery, School of Medicine, College of Medicine and Health Sciences, Mizan Tepi University, Mizan-Teferi, Ethiopia; c Department of Surgery, Gebre Tsadik Shawo General Hospital, Bonga, Ethiopia

## Abstract

**Introduction and importance:**

Gastric Outlet Obstruction (GOO) is a clinical syndrome due to mechanical obstruction of the gastric outlet near the antrum. The incidence of GOO is not known adequately; however, it is estimated that its incidence has declined in recent years as the incidence of peptic ulcer disease, which is the common cause of GOO, has been declining recently due to the use of proton pump inhibitor (PPI). The objective of this case report to highlight the importance of consideration of TB as a cause of GOO by affecting the duodenal wall and nearby lymph node enlargement**.**

**Case presentation:**

The case was a 31-year-old man who presented to the surgical referral clinic with a complaint of non-projectile vomiting of ingested matter. The patient also had a significant amount of weight loss. Laparotomy was done and displayed multiple enlarged pyloric and duodenal lymph nodes with a thickened duodenal wall. The patient was discharged from the ward after one week of hospital stay. For diagnosing the disease and relieving obstruction, laparotomy is usually required.

**Clinical discussion:**

Generally, gastric outlet obstruction is a common and early complication associated with duodenal ulcers. However, cases of gastric outlet obstruction caused by other factors are rare.

**Conclusion:**

In a patient presented with symptoms and signs suggestive of GOO with symptom complex of TB (tuberculosis). Early identification and appropriate management can lead to improved outcomes for patients with this rare form of tuberculosis.

## Hightlights

• Gastric Outlet Obstruction (GOO) is a clinical syndrome due to mechanical obstruction of the gastric outlet near the Antrum.
• Duodenal tuberculosis is one of the rare causes of GOO.
• It is rare to find a GOO in an adult caused by duodenal tuberculosis.
• There should be a high index of suspicion to consider Tuberculosis as a differential diagnosis for GOO in endemic area

## 1 Introduction

Gastric Outlet Obstruction (GOO) is a clinical syndrome due to mechanical obstruction of the gastric outlet near the antrum. A patient with GOO usually presents with abdominal pain in the epigastric area and vomiting following feeding [1]. The incidence of GOO is not known adequately; however, it is estimated that its incidence has declined in recent years as the incidence of peptic ulcer disease, which is the common cause of GOO, has been declining recently due to the use of PPI [2]. A 0.3 to 2.3 % of patients with tuberculosis may present with gastrointestinal (GI) involvement, and GOO is among the most common complications; however, GOO due to duodenal tuberculosis accounts for less than 2 % of cases [1]. Gastro-duodenal tuberculosis may mimic chronic peptic ulcer disease, gastric carcinoma, and periampullary tumor, which are the most causative agents of GOO [3]. A patient diagnosed with duodenal TB presents with signs and symptoms of GOO syndrome [3,4]. Duodenal TB results in GOO either due to extrinsic compression by resulting enlargement of nearby lymph nodes or the duodenal lumen obstruction by making the duodenal wall fibrosis; however, the simultaneous occurrence is rare [4,5]. In this case report, we reported a case of gastric outlet obstruction due to duodenal TB causing both fibrosis of the duodenal wall and lymph node enlargement in a 31-year-old patient to highlight the importance of consideration of TB as a cause of GOO by affecting the duodenal wall and nearby lymph node enlargement, which is unique to this case report, as it is not mentioned adequately in the textbook, despite. It can be treated with simple anti-TB medications [5]. This case report is in line with the SCARE Criteria [6].

## 2 Case report

The case was a 31-year-old man who presented to the surgical referral clinic with a complaint of non-projectile vomiting of ingested matter. It was non-bilious, occurred 5–6 times a day after feeding, contained undigested food matter, and had persisted for three years. The vomiting had progressively worsened over the previous six months. The patient also complained of a burning type of epigastric pain for which he was treated with PPI (proton pump inhibitor) on multiple occasions for the past three years; however, had no improvement. The patient also had a significant amount of weight loss. Despite these, the patient has no history of hematemesis, melena, or previous TB (tuberculosis) treatment. The patient appeared emaciated with a BMI (basal metabolic index) was 16 kg/m^2^; however, his vital signs were in normal range, and his conjunctiva was pale. On abdominal examination, a succession splash was appreciated; otherwise, no signs of peritonitis. A complete blood count showed anemia (Hg 7 g/dl); nevertheless, other parameters were in the normal range. ESR (erythrocyte sedimentation rate) was 30 mm/h and negative for HIV. Arterial Blood Gases (ABG) and serum electrolytes were not assessed as they were not available in the hospital.

**Fig. 1:**
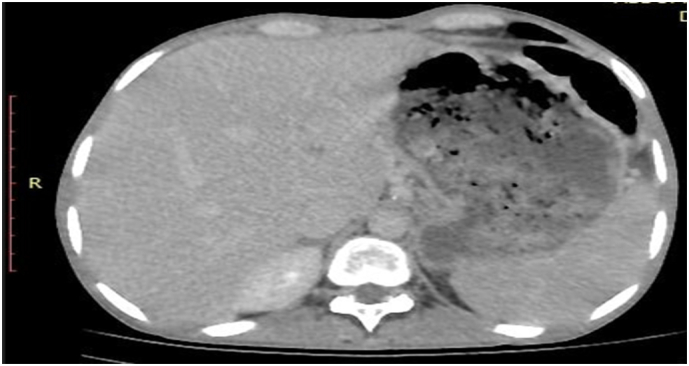
Abdominal CT scan in axil section which shows distended fluid filled stomach with outlet smooth tapering and multiple enlarged per pyloric lymph nodes.

**Fig. 2:**
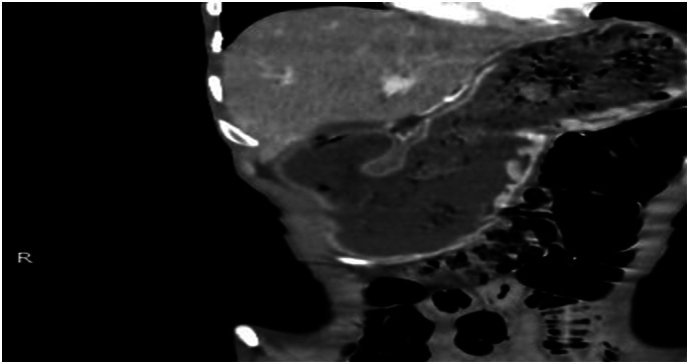
Abdominal CT scan in coronal section shows distended fluid filled stomach with outlet smooth tapering and multiple enlarged per pyloric lymph nodes.

**Fig. 3:**
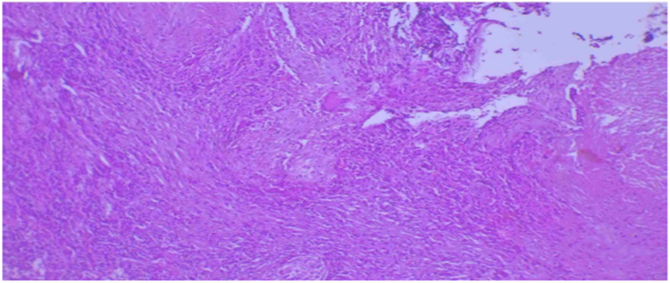
Lymph node biopsy which showed capsulated lymphoid tissue which is composed of heterogeneous lymphoid cells with germinal center formation with associated multinucleated giant cells and caseating epithelioid cell granuloma which is suggestive of Tuberculous Lymphadenitis.

A upper duodenum (Fig. 1 and Fig. 2); afterward, either upper GI endoscopy with biopsy or barium swallow was planned but not available. We entered the *peri*toneal cavity with a midline vertical abdominal incision; however, intra-operatively the stomach was grossly redundant, and there was multiple enlarged peri pyloric and per duodenal lymph nodes with associated fibrous tissue around the pylori-duodenal junction with thickened pylori-duodenal wall with cicatrization and stenosis of the pylori duodenal canal with decreased size of the duodenum due to associated scarring and fibrosis. Otherwise, there is no palpable gastric mass or periampullary tumor. There is no damage to the duodenum. So, we took two enlarged lymph nodes and dissected the lymph nodes, and there was a little milky discharge from the lymph nodes, so we suspected possible caseous necrosis that may be due to tuberculosis. So, we sent a lymph node biopsy and did a Gastrojejunostomy. Endoscopy (confirmatory for GOO) was not available in the nearby hospital; as a result, Contrast Abdominal CT was sent to confirm the diagnosis of Gastric Outlet Obstruction (GOO) and concurrent malignancy. The CT confirmed GOO secondary to chronic peptic ulcer diseases resulting from cicatrized A lymph node biopsy was taken for histopathologic examination and showed capsulated lymphoid tissue which is composed of heterogeneous lymphoid cells with the germinal center formation with associated multinucleated giant cells and caseating epithelioid cell granuloma which is suggestive of Tuberculous Lymphadenitis (Fig. 3).

The patient was discharged from the ward after one week of hospital stay and treated with anti-tuberculous drugs according to the national protocol for six months, and the above symptoms subsided without any post-operative or drug-related complications. The patient had gained 15 kg over two months at the follow-up visit and had no complaints. The wound site healed and no abnormal findings were appreciated.

## 3 Discussion

Gastric outlet obstruction refers to the blockage of the stomach specifically at the pyloroduodenal area. It occurs when there is scarring and swelling of the recurrent duodenal ulcer. In Africa, gastric outlet obstruction is a common and early complication associated with duodenal ulcers [6]. However, cases of gastric outlet obstruction caused by other factors are rare. This report focuses on tuberculosis as a cause of gastric outlet obstruction in the pyloroduodenum, even in the absence of clinical evidence of pulmonary tuberculosis, which has been frequently reported [7,8]. Tuberculosis, or TB, can affect any part of the gastrointestinal tract, including the mouth, anus, peritoneum, and pancreatobiliary system. It can have diverse symptoms, often resembling other common and uncommon diseases. Stomach and duodenal tuberculosis account for approximately 2 % of abdominal tuberculosis cases [2]. The most common site for gastrointestinal tuberculosis is the ileocecal region [9]. This may be due to factors such as reduced movement of materials, increased absorption of fluids and electrolytes, limited digestion, and a high concentration of lymphoid tissue in this area [10]. Consequently, chronic inflammation occurs and leads to significant thickening of the intestinal wall, causing narrowing of the lumen. Early involvement of regional lymph nodes can also occur, potentially leading to caseation in later stages [11].

A gastroduodenal location for tuberculosis is extremely uncommon, even in patients with pulmonary TB. An examination of autopsy records found that only 0.5 % of these patients had gastroduodenal TB lesions. Typically, gastroduodenal TB lesions are secondary in nature. Primary cases are quite rare, with only a few instances reported in the existing literature [12,13]. Isolated tuberculosis specifically affecting the duodenum is even more infrequent, likely due to the rapid passage of gastric contents through this section of the digestive system [3]. The rarity of duodenal involvement can be attributed to factors such as gastric acidity, the quick transit time of ingested organisms, the limited presence of lymph follicles in the gastric wall, and the intactness of the gastric mucosa [14]. From a clinical perspective, duodenal tuberculosis manifests as either gastric outlet or upper gastrointestinal obstruction. Diagnosis of this condition is typically achieved through endoscopic examination or laparotomy when complications related to intestinal obstruction arise [3]. Maintaining a high level of suspicion, coupled with radiological investigations, exploratory laparotomy, and histopathological examination of the tissue, can ultimately result in a definitive diagnosis of this uncommon condition [15].

Localized duodenal tuberculosis is an exceedingly rare condition, even in individuals with confirmed pulmonary tuberculosis. These individuals may exhibit symptoms akin to ulcers, and the initial evaluation of the upper gastrointestinal tract might reveal gastric outlet obstruction. Although the erythrocyte sedimentation rate (ESR) may be elevated, it was within normal range in our cases. It is not uncommon for these patients to be initially diagnosed and treated for peptic ulcers, which may lead to temporary relief. The largest documented series on duodenal tuberculosis originates from India and encompasses 30 cases. A significant majority (73 %) of these patients experienced symptoms related to duodenal obstruction. In the majority of these instances, the obstruction was a result of external compression by tuberculous lymph nodes, as was the case with our patients, rather than intrinsic lesions within the duodenum. The remaining cases (27 %) presented with a history of dyspepsia and were suspected to have duodenal ulcers [10].

The management approach for duodenal Tuberculosis primarily involves medical intervention, especially when a tissue diagnosis is available. The majority of patients show positive response to antituberculous treatment. For diagnosing the disease and relieving obstruction, laparotomy is usually required. Obstruction is commonly resolved through either resection or by-pass procedures. In cases of pyloro-duodenal obstruction, it is frequently necessary to perform gastro-enterostomy [9]. Following surgical treatment, it is crucial to administer a complete course of antituberculosis treatment [16]. Surgery is not recommended as the initial option in chronic and uncomplicated cases, as proper antituberculosis medication often leads to regression or disappearance of intestinal tuberculous lesions. In our case the diagnosis was settled post operatively and the patient was counseled for possible treatment option and possible outcomes before the surgery and preferred surgical option. The team also agreed as she presented with signs of obstruction and there was no endoscopy available in nearby setups.

## 4 Conclusion

Through the presentation of this case report, it is evident that maintaining a high level of suspicion for tuberculosis is crucial, especially in young individuals who present with gastric outlet obstruction or unresponsive or recurrent dyspepsia, particularly in regions where tuberculosis is prevalent. This case serves as an important reminder for healthcare professionals to remain vigilant for diverse patterns of extrapulmonary tuberculosis. Furthermore, individuals with extrapulmonary tuberculosis, such as gastroduodenal tuberculosis, may experience persistent symptoms of dyspepsia. Therefore, it is essential for physicians to recognize these symptoms as a potential indication of gastroduodenal tuberculosis. Early identification and appropriate management can lead to improved outcomes for patients with this rare form of tuberculosis.

## Authors' information

**MAK** is an Assistant Professor, Department of Medicine, School of Medicine, College of Medicine and Health Sciences at Mizan – Tepi University, Ethiopia. **MA** is a Medical Doctor and specialty certificate in Drug resistant tuberculosis management.

**SMM is** Assistant Professors of General Surgery, Department of Surgery, School of Medicine, College of Medicine and Health Sciences at Mizan – Tepi University, Ethiopia. SM is Medical Doctors and has Specialty Certificate in General Surgery.

**DWA** is a General Practitioner in Department of Surgery, School of Medicine, College of Medicine and Health Sciences at Mizan – Tepi University, Ethiopia. DW is a Medical Doctor.

**YTN is** a General Surgeon, Department of Surgery, Gebre Tsadik Shawo General Hospital, Bonga, Ethiopia. YT is Medical Doctors and has Specialty Certificate in General Surgery.

WT and ZG.

**ZGT** and **WMT** are a General Practitioner, Department of Medicine, School of Medicine, College of Medicine and Health Sciences at Mizan – Tepi University, Ethiopia. **MA** is a Medical Doctor.

## Informed consent

Before preparing the case report, the patient provided written, informed consent to write and publish the case report.

## Ethical approval

The case report was submitted for Ethical Review Committee and approved as ethically correct.

## Funding

The case report, authorship, and/or publication of this work were done without any funding.

## Author contribution

Molla Asnake Kebede, MD. Involved in the conception and design of the study, drafting and revising of the article and final approval of the version to be submitted and also involved in direct management of the patient.

Sisay Mengistu Mohamed, MD. Involved in the conception and design of the study, drafting and revising of the article and final approval of the version to be submitted and also involved in direct management of the patient.

Dessalegn Woretaw Ayehu, MD. Involved in the design of the study, drafting and revising of the article and final approval of the version to be submitted.

Yilkal Teshome Numaro, MD. Involved in the design of the study, drafting and revising of the article and final approval of the version to be submitted.

Woineab Mengista Tadeg, MD. Involved in the design of the study, drafting and revising of the article and final approval of the version to be submitted.

Zerubabel Girma Tesso, MD. Involved in the design of the study, drafting and revising of the article and final approval of the version to be submitted.

All authors agreed to be accountable for all aspects of the manuscript.

## Guarantor

Molla Asnake Kebede.

## Research registration number

Not applicable.

## Conflict of interest statement

No potential conflicts of interest were disclosed by the author(s) with regard to the case report, writing, or publication of this article.

## Data Availability

On a valid request, the corresponding author will provide access to the datasets that were gathered and used to conduct this article.
